# Role of prolactin receptor and CD25 in protection of circulating T lymphocytes from apoptosis in patients with breast cancer

**DOI:** 10.1038/sj.bjc.6600860

**Published:** 2003-04-15

**Authors:** T Bauernhofer, I Kuss, U Friebe-Hoffmann, A S Baum, G Dworacki, B K Vonderhaar, T L Whiteside

**Affiliations:** 1 University of Pittsburgh Cancer Institute, Pittsburgh, PA 15213-1863, USA; 2 Basic Research Laboratory, National Cancer Institute, NIH, Bethesda, MD 20892, USA; 3 Department of Pathology, University of Pittsburgh School of Medicine, Pittsburgh, PA 15213-1863, USA; 4 Department of Psychiatry, University of Pittsburgh School of Medicine, Pittsburgh, PA 15213-1863, USA

**Keywords:** apoptosis, prolactin, Annexin V, Fas, prolactin receptor

## Abstract

Prolactin (PRL) has been reported to inhibit apoptosis in various cell types and to serve as a cofactor in the upregulation of CD25 on T cells during activation. We investigated a possible relation between prolactin receptor (PRL-R) or IL-2 receptor alpha (IL-2R*α*, CD25) expression on circulating T lymphocytes and their apoptosis in patients with breast cancer. Peripheral blood mononuclear cells obtained from 25 patients, 25 normal controls (NC) and three cord blood samples were evaluated for Annexin V binding and expression of CD95, CD25, and PRL-R on CD3^+^ T cells by multicolour flow cytometry. Plasma levels of PRL, sCD95L, and sIL-2R were determined in patients and controls and related to T-cell apoptosis. The ability of PRL to protect T cells from apoptosis induced by various agents was also studied. Expression of PRL-R on the surface of T cells was comparable in patients with breast cancer and NC, but PRL plasma levels in patients were significantly lower (*P*<0.05). In patients, 18±11% (mean±s.d.) of CD3^+^ cells bound Annexin V, compared to 9±6% in NC (*P*<0.0004). Percentages of CD3^+^Fas^+^ and CD3^+^CD25^+^ cells were higher in the peripheral circulation of patients than NC (*P*<0.0001 and <0.04, respectively). Levels of sFasL were lowest in plasma of the patients with the highest proportions of CD3^+^Fas^+^ T cells. Most T cells undergoing apoptosis were CD3^+^CD25^−^ in patients, and the proportion of CD3^+^CD25^−^ Annexin V^+^ cells was significantly increased in patients compared to NC (*P*<0.006). *Ex vivo* PRL protected T cells from starvation-induced or anti-CD3Ab-induced but not from Fas/FasL-dependent apoptosis. These results indicate that expression of CD25 but not of PRL-R on the surface of activated T lymphocytes appears to be involved in modulating Fas/Fas – ligand interactions, which are, in part, responsible for apoptosis of T lymphocytes and excessive turnover of immune cells in the circulation of patients with breast cancer.

Patients with cancer often have dysfunctions of the immune system (Kiessling *et al*, 1999; Whiteside, 2001). We have shown previously that the proportion of circulating T lymphocytes undergoing apoptosis is significantly higher in patients with melanoma or head and neck cancer compared to healthy individuals (Saito *et al*, 2000; Dworacki *et al*, 2001; Hoffmann *et al*, 2002). Decreased or absent ζ chain expression in T lymphocytes and elevated percentages of Fas^+^CD3^+^ lymphocytes in the peripheral circulation of patients with solid tumours have been linked to the increased susceptibility of their T lymphocytes to ‘spontaneous’ apoptosis (Kuss *et al*, 1999; Chen *et al*, 2000; Dworacki *et al*, 2001; Hoffmann *et al*, 2002). However, there is relatively little information available about the mechanisms underlying these phenomena in cancer patients or about factors that could protect the immune cells from spontaneously undergoing programmed cell death.

Several lines of evidence demonstrate that prolactin (PRL), a peptide hormone, which is synthesised and secreted primarily by the anterior pituitary gland but also produced in many extrapituitary sites, including breast tissue and T lymphocytes, is an important immunoregulatory factor (Matera, 1996; Bole Feysot *et al*, 1998). Prolactin modulates functions of the immune system by stimulating cell proliferation and prolonging cell survival (Clevenger *et al*, 1998). Several *in vitro* studies have suggested that exogenous PRL can act as a comitogen for Concanavalin A (Con A)-stimulated splenocytes (Montgomery *et al*, 1987), IL-2-stimulated L2 T cells (Clevenger *et al*, 1990) and human peripheral blood lymphocytes (Matera *et al*, 1992). In rat Nb2 T cells, PRL has been shown to act as a mitogen (Yu Lee, 1990). Porlactin effects are mediated by the prolactin receptor (PRL-R), a member of the haematopoietin cytokine receptor superfamily (Bazan, 1989), which is ubiquitously expressed on immune cells (Dardenne *et al*, 1994).

The immunomodulatory effects of PRL may be owing to its ability to induce directly the expression of growth-related genes (Yu Lee, 1990). Alternatively, PRL could act indirectly by upregulation of the expression of cytokine receptors, such as IL-2 receptor alpha (IL-2R*α*, CD25) as reported for rat splenocytes (Mukherjee *et al*, 1990) and for mouse CD4^+^ and CD8^+^ T cells (Gala and Shevach, 1993). Prolactin could also upregulate expression of its own receptor on human peripheral blood lymphocytes (Dardenne *et al*, 1994), including T cells (O'Neal *et al*, 1991). On the other hand, IL-2 is known to be able to modify the PRL/PRL-R pathway in T lymphocytes: upon IL-2 activation of T cells, a rapid translocation of PRL from the cell surface into the nucleus and of the PRL-R to the nuclear periphery is observed (Clevenger *et al*, 1990). One of the less known effects of PRL is the ability to inhibit apoptosis in various cell types, including the Nb2 rat lymphoma cell line (LaVoie and Witorsch, 1995; Weimann *et al*, 1999). In addition, evidence exists that human PRL-antagonists, such as HPRL-G129R, can inhibit cancer cell proliferation through induction of apoptosis (Chen *et al*, 1999).

Current evidence suggests that a close relation exists between PRL-R, IL-2R, and their ligands, which influences cell growth, activation, and death of immune cells. In this paper, we examine the possibility that the PRL/PRL-R is involved in protection of CD3^+^ immune cells from apoptosis in patients with breast cancer. To this end, PRL-R expression on T cells and levels of serum PRL were examined and correlated with the Annexin V binding and expression of CD95 and CD25 on these cells in the peripheral circulation of women with breast cancer and healthy controls.

## MATERIALS AND METHODS

### Patients

Peripheral blood mononuclear cells (PBMC) were obtained from 25 women with breast cancer, who were followed either at the Outpatient Clinic of the University of Pittsburgh Cancer Institute or at Magee Womens Hospital. Peripheral blood mononuclear cells were also obtained from 25 healthy volunteers as well as three cord blood samples. The patients and healthy volunteers enrolled in this study all had signed informed consent forms approved by the IRB and the IRB for Magee Womens Hospital. Patients included in this study all had previous surgery and/or other therapies for breast cancer. They were evaluated at least 3 months (median 13 months) after the termination of either postoperative radiation and/or chemotherapy. Some patients (*n*=3) and healthy controls (*n*=5) were studied at more than one time point. The patients and controls were sex- and age-matched, with a mean age±s.d. of 60±7 years for the breast cancer patients and 54±15 years for the control group. Patient characteristics are listed in Table 1

**Table 1:** Clinicopathological characteristics of the patients with breast cancer included in the study

**Characteristics**	**Patients (n=25)**
*Age*	
median (range; years)	63 (45–76)
*Tumor stage at the time of diagnosis*	
Stage 0	2
Stage 1	15
Stage 2	6
Stage 3	2
*Prior treatment*^a^	
Surgery^1^	25
Postoperative XRT^2^	18
Chemotherapy^3^	14
Chemotherapy and XRT^4^	8
*Disease status at time of blood draw*	
M0	23
M1	2
*Treatment at time of blood draw*	
Hormonal therapy (tamoxifen)	11

a Termination of prior treatment in months (median) before time of blood draw:

1 27;

2 16;

3 13.5;

4 13.

.

### Peripheral blood mononuclear cells

Venous blood (20 ml) drawn in lithium heparin tubes was obtained from the patients and controls. Peripheral blood mononuclear cells were isolated by Ficoll – Hypaque gradient centrifugation, washed twice in Dulbecco's phosphate-buffered saline (D-PBS; Life Technologies, Inc., Grand Island, NY, USA), counted in a trypan blue dye, and immediately used for experiments. The time elapsed between venipuncture and testing was about 3 h. The plasma was aliquoted into vials and stored at −80°C for testing of PRL levels.

### Cell lines

The human breast cancer cell line T47D and the Jurkat human T-cell lymphoma cell line were obtained from the ATCC (Manassas, VA, USA). The two cell lines were maintained in RPMI 1640 medium (Life Technologies) supplemented with 10% (v v^−1^) foetal calf serum (FCS), 1 mM glutamine, 100 U ml^−1^ penicillin, and 100 *μ*g ml^−1^ streptomycin (all from Life Technologies, Inc.). The T47D cell line was passaged by trypsinisation. Before staining for PRL-R, T47D cells were detached from plastic using a cell dissociation solution (Sigma, St Louis, MO, USA). Tests for mycoplasma (Gen Probe, San Diego, CA, USA) were performed at monthly intervals and were negative for all cell lines.

### Staining of cells for flow cytometry

#### Prolactin receptor

Suspensions of PBMC were prepared containing 5 × 10^5^ cells per 100 *μ*l of D-PBS washing buffer supplemented with 0.1% (w v^−1^) sodium azide, and 0.1% (w v^−1^) bovine serum albumin (BSA). The cell suspensions were incubated for 30 min at 4°C with 1 *μ*l of pretitred stock solution (1 mg ml^−1^) of unlabelled, protein-A purified mouse anti-human PRL-R monoclonal antibody (mAb), B6.2 (IgG1) described previously (Banerjee *et al*, 1993). Mouse IgG1 (DAKO Corp., Carpinteria, CA, USA) was used as an isotype control. Erythrocytes were then lysed with NH_4_CL, and the cells were washed twice and incubated with FITC-conjugated goat anti-mouse IgG1 F(ab)_2_ (GAMIg, Caltag, San Francisco, CA, USA) for 30 min at 4°C. In chessboard titrations, the optimal concentration was found to be 10 *μ*g ml^−1^ for the first-step mAb, B6.2, and 50 *μ*l ml^−1^ for the second-step mAb, GAMIg. After two washes, an excess of mouse IgG1 was added to PBMCs for 15 min at room temperature (RT) to saturate free binding sites on the FITC-conjugated secondary antibody and to prevent nonspecific staining. The cells were then incubated with one or two of the following phycoerythrin (PE)- or PerCP-conjugated anti-human mAbs: anti-CD3, anti-CD56, or the appropriate IgG1 isotype control (all purchased from Becton Dickinson, San Jose, CA, USA) for 30 min at 4°C. After two washes, the cells were fixed with 0.5% (w v^−1^) paraformaldehyde in D-PBS. All mAbs were pre-titred on normal PBMCs to determine their optimal dilutions.

In all experiments, cells of each patient were always tested in parallel with those obtained from a normal control. The T47D breast cancer cell line was used as a positive control.

#### Annexin V, CD95, and CD25

Fresh PBMC were washed once in buffer, divided into aliquots of 5 × 10^5^ cells in 100 *μ*l of the same buffer and incubated in the presence of PE-labelled CD95 mAb (Clone DX2, PharMingen, San Diego, CA, USA) or PE-labelled anti-CD25 mAb, PerCP-labelled anti-CD3 mAb or respective isotype control mAbs (all from Becton Dickinson) for 30 min at 4°C. After one wash with buffer and with Annexin V-buffer (PharMingen), the cells were resuspended in 100 *μ*l of Annexin V-buffer and incubated with 1 *μ*l of the AnnexinV-FITC conjugate (PharMingen) for 20 min at RT in the dark. Next, an aliquot of 300 *μ*l Annexin V-buffer was added to each tube, and the flow cytometry analysis was performed within the next 60 min.

### Flow cytometry

Three-colour flow cytometry analysis was performed on a FACScan (Becton Dickinson, Mountain View, CA, USA) equipped with a single 488 nm argon ion laser. For each sample, at least 10 000 events were acquired. The amplification and compensation were set according to a standard procedure used in our laboratory and described (Dworacki *et al*, 2001; Hoffmann *et al*, 2002). For the PRL-R determination in PBMC, gates were set on lymphocytes and backgated on a CD3^+^/CD56^−^ cell population to establish the relative cell number and arithmetric mean fluorescence intensity (MFI). The percentage of apoptotic cells was calculated by scoring Annexin V-binding cells after backgating on CD3^+^ cells in the third colour. All gated mononuclear cell subpopulations were visualised on forward angle scatter/side angle scatter (FSC/SSC) dot-plots. In order to include all apoptotic cells and avoid debris with a high SSC signal, the gate was set to include a wide boundary of mononuclear cells (‘open gate’), since apoptotic cells accumulated mainly in the lower FSC/SSC channels (Darzynkiewicz *et al*, 1992; Saito *et al*, 2000; Dworacki *et al*, 2001). The cutoff for apoptotic cells was set based on staining of PBMC pretreated with CH-11 Ab (200 ng ml^−1^; UBI Biotechnology, Lake Placid, NY, USA), using Annexin V-positive cell population and Annexin V plus PI-positive population. In the subsequent experiments, apoptotic cells (Annexin V^+^) were quantitated within the CD3^+^CD25^+^ or CD3^+^CD25^−^ as well as CD3^+^CD95^+^ and CD3^+^CD95^−^ cell populations by three-colour flow cytometry.

### Induction of apoptosis in Jurkat cells

Jurkat cells (1 × 10^6^ ml^−1^) were suspended in RPMI 1640 medium supplemented with 0.5% charcoal-striped FCS for 24 h prior to the experiments. Apoptosis was induced either by further serum starvation or by incubation in the presence of CH-11 Ab (200 ng ml^−1^), 20 mM VP-16 (Sigma) or 100 nM dexamethasone (Sigma) with or without the addition of human PRL (20–200 ng ml^−1^, a gift of Dr Parlow, NHPP, UCLA, Torrance, CA, USA) for 2, 4, 6, and 24 h at 37°C. The time course of Annexin V binding to Jurkat cells was established by flow cytometry performed as described above.

### Activation of PBMC with anti-CD3Ab, IL-2, and PRL

Wells of 96-well plates were coated with anti-CD3Ab (0.5 *μ*g ml^−1^) in D-PBS at 37°C for 90 min and washed three times with D-PBS. Peripheral blood mononuclear cells obtained from normal donors were seeded at the concentration of 4 × 10^5^ cells per 200 *μ*l of AIM-V medium into each well. Either IL-2 at the concentration of 25 IU ml^−1^ or PRL at the concentration of 20 or 200 ng ml^−1^ or the combination of IL-2 and PRL were added to the wells. Wells not coated with anti-CD3Ab and not containing IL-2 or PRL served as controls. T-cell activation experiments were performed in an atmosphere of 5% CO_2_ in air at 37°C for 72 h. For proliferation assays, 1 *μ*Ci of ^3^H-labelled thymidine was added to each well 18 h before harvesting the cells. Alternatively, the cells were harvested, washed twice with buffer and stained for Annexin V, PI, CD95, CD25, CD71, and CD3 as described above.

### Measurements of PRL, soluble Fas Ligand (sFasL), and soluble IL-2 receptor (sIL-2R) in plasma

Blood was drawn between 9 and 12 a.m. from all patients and controls. Plasma was recovered from whole blood of the sample collected on the same day as that used for lymphocyte testing after centrifugation for 10 min at 2000 r.p.m. and was frozen immediately at −80°C. All tests were performed in a single assay. A two-side sandwich immunoassay to measure PRL levels was performed at the Clinical Chemistry Laboratory, UPMC. This chemoluminescence assay uses constant amounts of two Abs, a polyclonal goat-anti-human PRL Ab labelled with acrothymium ester and a mouse-anti-human PRL mAb covalently coupled to paramagnetic particles, for detection of PRL. A direct relation exits between PRL present and the amount of relative light units (RLU) detected by Advia-Centaur analyzer (Bayer, Pittsburgh, PA, USA), and the RLU are automatically translated into PRL ng ml^−1^ by the analyzer.

Soluble FasL was determined by a quantitative sandwich enzyme immunoassay (Oncogene Research Products, Boston, MA, USA), employing a mAb specific for the human FasL protein. The lower limit of detection for sFasL was 0.02 ng ml^−1^.

Soluble IL-2R was quantitated using an enzyme-linked immunosorbent assay (Cellfree® human ELISA sIL-2R, Pierce-Endogen, Rockford, IL, USA). The assay sensitivity was <24 U ml^−1^ sIL-2R.

The procedures for both assays were carried out according to the protocol provided by the manufacturers.

## STATISTICAL ANALYSES

Unpaired two-tailed *t*-test was used to evaluate differences between breast cancer patients and normal controls. Linear regression was employed to examine correlations between all the parameters studied and age. No correlation of any of the parameters investigated in this study with age was observed in either patients or controls. Graphpad Instat software (Version 3.01 for Windows 95/NT, GraphPad Software, San Diego, CA, USA) was used for all statistical analyses performed.

## RESULTS

### Prolactin and apoptosis induction in Jurkat cells

**Figure 1:**
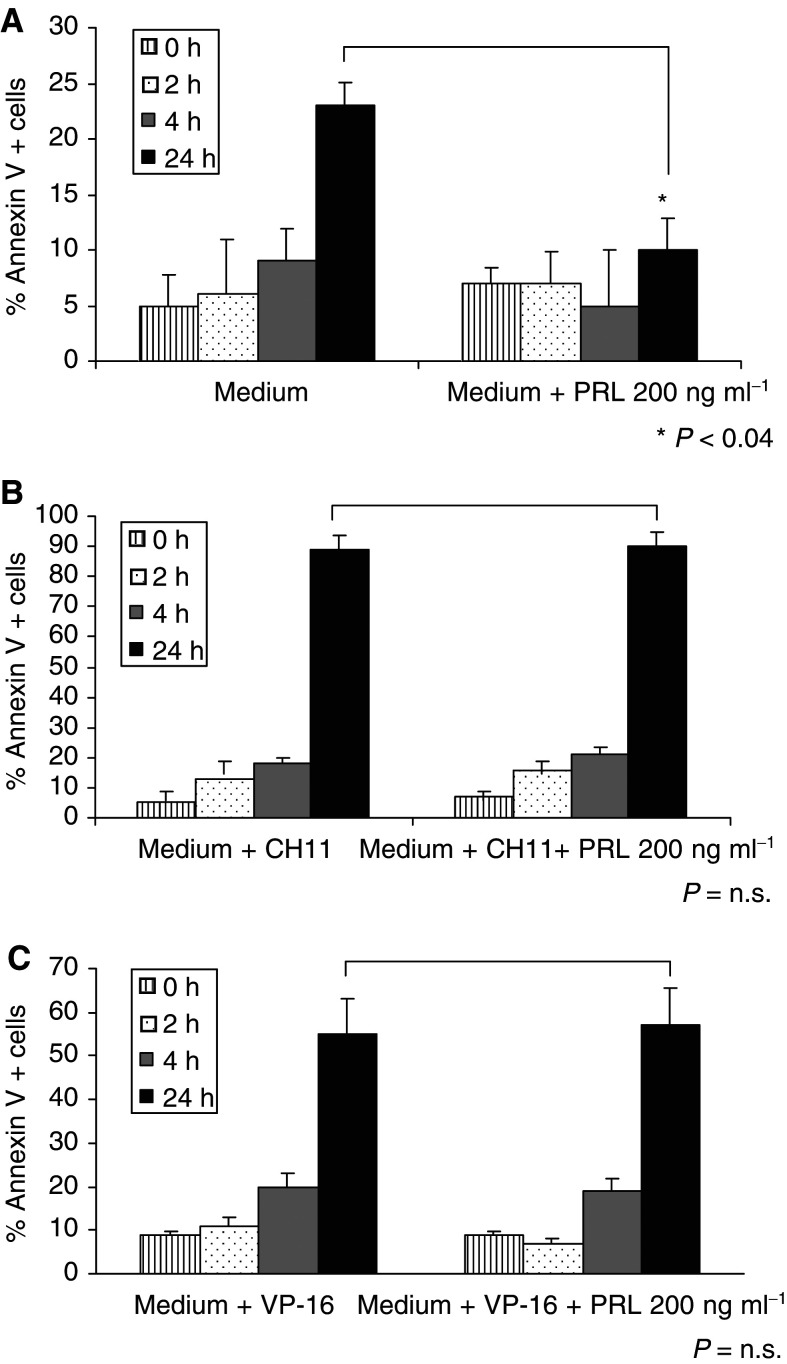
*Ex vivo* protection of Jurkat cells from apoptosis by exogenous PRL. Jurkat cells were incubated in the presence or absence of 200 ng ml^−1^ of PRL (**A**); 200 ng ml^−1^ of CH-11 Ab (**B**); or 20 mM VP-16 (**C**). Following incubation for various periods of time, Annexin V binding to Jurkat cells was measured by flow cytometry as described in Materials and Methods. The data are presented as means ±s.d. A representative experiment of two performed is shown.

In order to test the hypothesis that PRL might be able to inhibit the onset of apoptosis in T lymphocytes, the Jurkat cell line was used as a model system. The rationale for selecting Jurkat cells, instead of PRL-dependent Nb-2 rat lymphoma cell line, was to have a human model system previously shown to be PRL sensitive (Matera *et al*, 1997). Apoptosis was induced in Jurkat cells either by starvation in RPMI 1640 medium supplemented with 0.5% charcoal-striped FCS or by treatment of the cells with the Fas crosslinking CH-11 mAb, dexamethasone, or VP-16 for various time periods (Figure 1

). The apoptosis-inducing agents were added in the presence or absence of different PRL concentrations (data not shown). Prolactin at the concentration of 200 ng ml^−1^ was found to significantly decrease apoptosis induced by serum starvation (Figure 1A). However, PRL did not prevent the onset of apoptosis in Jurkat cells treated with CH-11 mAb or VP-16 (Figure 1B, C). Jurkat cells turned out to be insensitive to apoptosis induced by corticosteroids, and it was not possible to evaluate effects of PRL on dexamethasone-induced apoptosis. Instead, our results showed that in the presence of dexamethasone, PRL was no longer able to prevent starvation-induced apoptosis (data not shown), confirming previously reported antagonistic effects of dexamethasone on PRL activity (Clevenger *et al*, 1998).

### Prolactin receptor expression of T lymphocytes and PRL plasma levels

**Figure 2:**
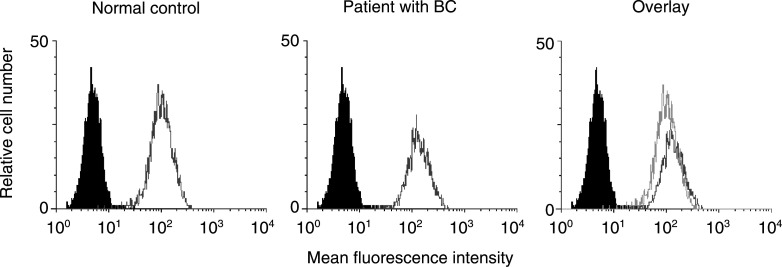
A series of flow cytometry histograms showing the expression of the PRL-R on CD3+ T cells (▪, Isotype control, □, Prl-R+ CD3 cells). Virtually all CD3+ T cells are PRL-R positive on the cell surface in PBMC of a typical patient with breast cancer (BC) and a normal control. Three-colour flow cytometry was performed as described in Materials and Methods.

To evaluate the role of PRL and PRL-R in spontaneous apoptosis of circulating T cells observed in cancer patients, we measured these parameters in patients with breast cancer and in normal controls. By flow cytometry, all circulating CD3^+^ T lymphocytes were positive for PRL-R expression in breast cancer patients as well as control individuals. The MFI of the PRL-R on T lymphocytes of breast cancer patients was 56–175 (median 106) compared to 60–176 (median 101) in normal controls, indicating a similar level of PRL-R expression on T lymphocytes in patients and controls (Figure 2

). Annexin V binding to CD3^+^ T cells was then evaluated in the same cohorts of patients and controls. As shown in Table 2

**Table 2:** Proportions of CD3^+^ T cells expressing CD95 and binding Annexin V in PBMC and plasma levels of sCD95L in patients with breast cancer, normal controls and cord blood

	**Annexin V^+^ CD3^+^(%)**	**CD95^+^ CD3^+^ (%)**	**Annexin V^+^ CD95^+^ CD3^+^ (%)**	**sCD95L in plasma (ng ml^−1^)**
Patients (*n*=25)	18±11	67±16	15±11	0.2±0.2
Normal controls (n=25)	9±6	47±11	7±5	0.4±0.3
Cord blood (*n*=3)	1±2	4±2	0.5±0.5	1.9±0.3
*P*^a^	0.0004	0.0001	0.0003	0.007

a The data are means±s.d. The *P*-values refer to the difference between the means of normal controls and patients with breast cancer.

, a significantly higher proportion of T cells was undergoing apoptosis in the peripheral circulation of patients than of controls. Thus, despite the equivalent expression of PRL-R on all circulating T cells in patients and controls, spontaneous apoptosis was significantly greater (*P*<0.0004) in the former.

Since there was a possibility that levels of PRL in the circulation could influence PRL-R signalling, we next quantitated plasma levels of PRL in the patients and controls. We found that PRL plasma levels were significantly lower in patients compared to normal controls (5.7±3 *vs* 8.2±4 ng ml^−1^; *P*<0.05). However, no correlation could be established between Annexin V binding, signifying the onset of early apoptosis, and PRL-R expression or PRL plasma levels. In addition, CD25 expression on T cells did not correlate with either of these parameters.

### Fas (CD95) expression and Annexin V-binding to T lymphocytes

**Figure 3:**
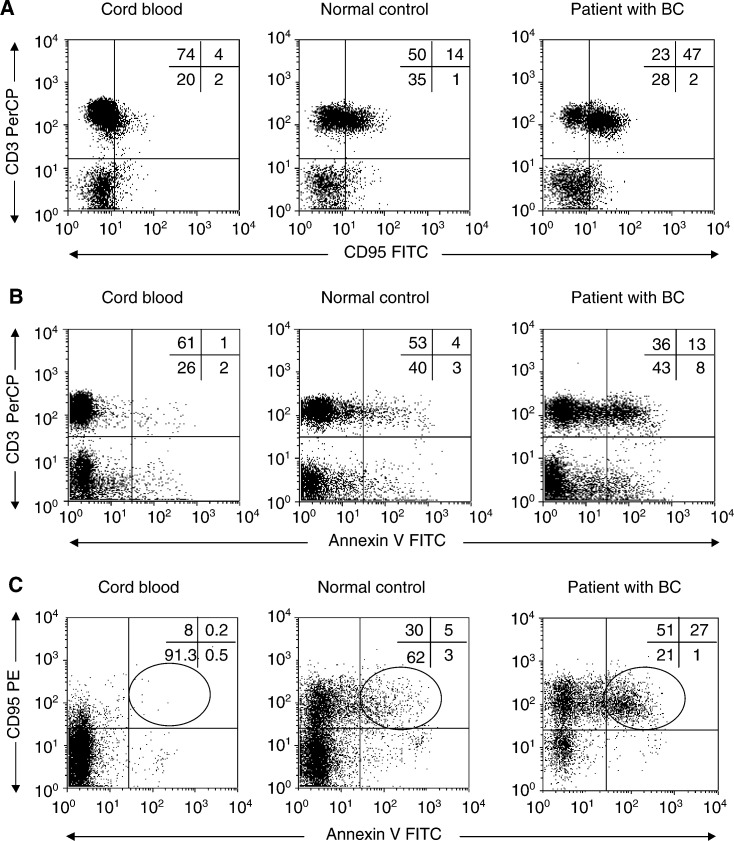
Expression of CD95 on CD3^+^ T cells (**A**); Annexin V binding to CD3^+^ T cells (**B**) and Annexin V binding to CD95^+^ cells (**C**) in PBMC obtained from a representative patient with BC, a normal donor, or cord blood. Backgate was set on CD3^+^cells. Three-colour flow cytometry was performed as described in Materials and Methods.

In our previous studies of patients with cancer, CD95^+^CD3^+^ T cells accounted for a major proportion of circulating lymphocytes (Hoffmann *et al*, 2002). Therefore, flow cytometry was used to determine the proportion of circulating CD95^+^ T lymphocytes in patients with breast cancer and controls. As shown in Table 2, the proportion of CD95^+^CD3^+^ cells was significantly elevated in patients (*P*<0.0001) relative to controls. Very few cord blood T cells expressed Fas. Since Fas expression on T cells could be responsible for their susceptibility to apoptosis, we next determined the proportion of Annexin V-binding Fas^+^ T cells in the same specimens. A significantly higher percentage of Fas+ T cells bound Annexin V in patients than in controls (*P*<0.0003) and in cord blood, only 0.5±0.5% of T cells were Fas^+^Annexin V^+^ (Table 2). Representative flow cytometry data for a patient with breast cancer, a control donor, and a cord blood sample are shown in (Figure 3A – C

). The results clearly show that in patients with breast cancer, Fas^+^CD3^+^ T cells preferentially bind Annexin V.

Analyses were next performed to determine whether the extent of spontaneous T-cell apoptosis could be related to the disease stage, presence of metastases, or previous therapy in patients with breast cancer. Interestingly, even in patients with Stage I disease at the time of diagnosis and no evidence of recurrence at the time of the blood draw, we observed a significantly higher percentage of circulating T cells binding Annexin V (Table 3

**Table 3:** Proportions of T lymphocytes expressing CD95 and binding Annexin V in PBMC of patients with breast cancer relative to their clinicopathological features, disease stage, and prior or concurrent treatments

	**CD95+ CD3+ (%)^a^**	**Annexin V+ CD3+ (%)^a^**	**Annexin V+ CD95+ CD3+ (%)^a^**
All patients (*n*=25)	67±16	18±11	15±11
M0 (*n*=23)	70±16	18±10	15±10
M1 (*n*=2)	87±4	35±10	35±10
Stage 1 (*n*=15)^b^	66±16	16±8	14±7
Stage > 1 (*n*=8)	76±16	25±10	20±11
No XRT (*n*=7)	75±15	13±9	11±8
XRT (*n*=18)	65±17	19±11	16±11
No Chemo (*n*=11)	63±17	17±13	15±12
Chemo (*n*=14)	75±16	19±10	17±10
Prior XRT & Chemo (*n*=8)	77±17	22±10	19±10
No Tamoxifen (*n*=14)	68±20	19±13	15±14
Tamoxifen (*n*=11)	65±11	17±6	14±6

a The data are means±s.d. XRT=radiation therapy.

b Two patients with Stage 0 are not included in this analysis.

) as compared to normal controls. Also, prior postoperative radiation or adjuvant chemotherapy did not alter the proportions of apoptotic T cells in breast cancer patients. It is important to note that the data for Annexin V binding and CD95 expression were highly consistent in those normal individuals who were tested more than once. It was possible to confirm in some cases that the proportions of CD95^+^, Annexin V^+^, or CD95^+^Annexin V^+^ T cells in the peripheral circulation remained unchanged over a time period of up to 1 year. In contrast, proportions of Annexin V^+^CD95^+^CD3^+^ T cells were less stable in patients with breast cancer, as two out of four patients for whom two serial tests were available had a decreased and the other two increased proportions of these cells on repeated examination.

### Soluble FasL (sCD95L) plasma levels in patients and controls

**Figure 4:**
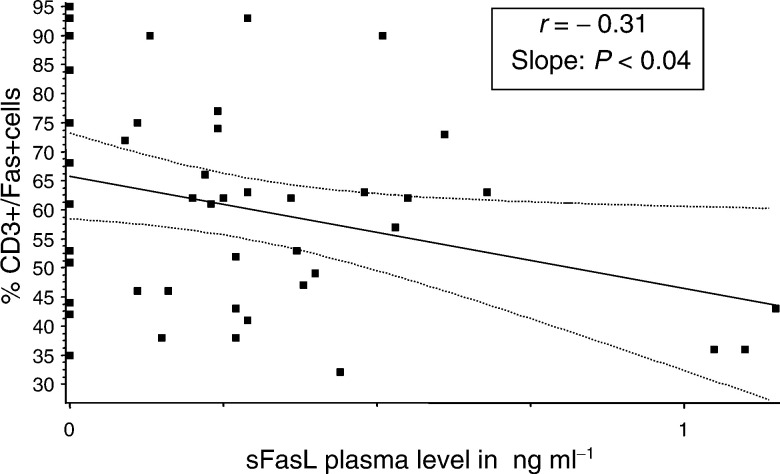
Correlation between sFasL in plasma and expression of Fas on CD3^+^ T cells in the patients with BC and normal controls. The *P*-value shown is for combined analysis of patients and controls. When cord blood samples were included, the *P*-value was 0.0001.

In order to attempt to explain the mechanisms responsible for the higher onset of early apoptosis in CD95^+^ T cells in patients with breast cancer, we next determined plasma levels of sCD95L in the cohorts of patients and controls. The expectation was that sCD95L might be utilised in patients through its binding to CD95 expressed on T cells. Indeed, a statistically significant lower level of sCD95L was found in patients compared to normal controls and cord blood, as shown in Table 2. Those breast cancer patients with the highest percentage of CD95^+^ CD3^+^ T cells had the lowest sCD95L levels in the plasma (Figure 4

), suggesting increased binding or utilisation of the sCD95L in these individuals. These results imply that sCD95L might contribute to apoptosis of CD3^+^Fas^+^ lymphocytes in the circulation of patients with breast cancer.

### IL-2 R*α* (CD25) expression and Annexin V binding to T lymphocytes

Similar to Fas expression, CD25 expression is considered to be a result of T-cell activation. We, therefore, examined CD25 expression on T cells that bind Annexin V in a subgroup of patients and an age-matched subgroup of normal individuals as well as in cord blood samples (Table 4

**Table 4:** Proportions of CD25^+^CD3^+^Annexin V^+^ lymphocytes and MFI of CD25 expression in cord blood and in PBMC of normal controls and patients with breast cancer

	**CD25^+^ CD3^+^ (MFI)^a^**	**Annexin V^+^ CD25^+^ CD3^+^ (%)^a^**	**Annexin V^+^ CD25^−^ CD3^+^ (%)^a^**	**sIL-2R*α* (U ml^−1^)**
Patients (*n*=18)	233±26	16±3	19±11	761±285
Normal controls (*n*=18)	206±23	10±5	8±6	488±166
Cord blood (*n*=3)	109±13	4±2	1±1	827±162
*P*^b^	0.02	0.08	0.006	0.01
^a^The data are means±s.d. MFI=mean fluorescence intensity; CD25=IL-2R*α*; sIL-2R*α*=soluble IL-2R*α*.				
^b^The *P*-values reflect comparisons performed between normal controls and breast cancer patients.				

). We observed a statistically significant increase in the percentage of CD25^+^ T cells (*P*<0.04) as well as in the MFI for CD25 (*P*<0.02) in CD3^+^ lymphocytes of patients as compared to normal controls. These findings are consistent with the observed significant increase in the level of sIL-2R in plasma of the patients (Table 4). An increased proportion of CD3^+^ lymphocytes in patients was found to be Annexin V^+^CD25^+^ as compared to controls (NSD, Table 4). Unexpectedly, however, there was a highly statistically significant difference in the percentage of CD3^+^ CD25^−^ cells that bound Annexin V in patients as compared to controls (*P*<0.006). These results suggested that CD3^+^ CD25^−^ T cells were also targeted for apoptosis in the circulation of patients with breast cancer. Representative data for the CD25 expression and Annexin binding by T cells in a patient with breast cancer, a normal donor, and a cord blood sample are shown in Figure 5

**Figure 5:**
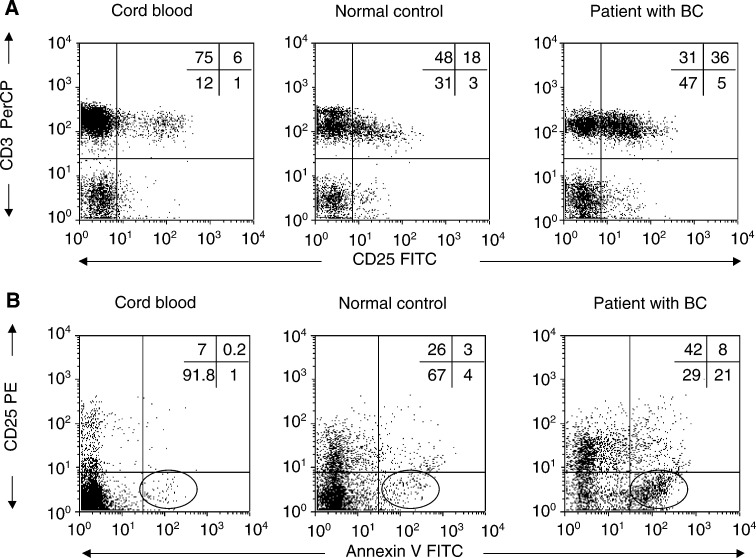
Expression of CD25 on CD3^+^ T cells (**A**) and Annexin V binding to CD25^+^ cells (**B**) in PBMC obtained from a representative patient with BC, a normal donor, or cord blood. Backgate was set on CD3^+^ cells. Three-colour flow cytometry was performed as described in Materials and Methods.

. Importantly, these data demonstrate that in patients, CD25^−^ T lymphocytes preferentially bind Annexin V (Figure 5B).

### *Ex vivo* T-cell activation with anti-CD3Ab or IL-2 in the presence or absence of PRL

**Figure 6:**
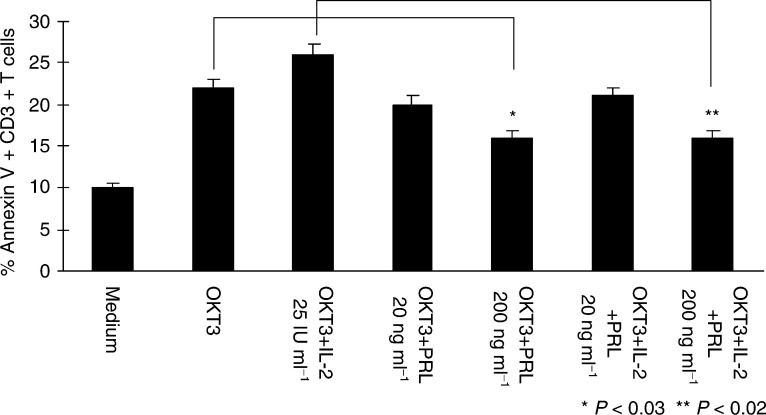
*Ex vivo* activation of normal PBMC with anti-CD3Ab (0.5 *μ*g ml^−1^) in the presence or absence of PRL and/or IL-2. After 72 h of incubation, the proportion of Annexin V-binding CD3+ T cells present in culture was determined by multicolour flow cytometry. Representative data from two experiments preformed are presented as means±s.d.

To address the question of whether the state of T-cell activation could be related to the higher proportions of T lymphocytes undergoing apoptosis, as seen in patients with breast cancer, we performed *ex vivo* experiments, using plastic immobilised anti-CD3Ab for lymphocyte activation. When normal PBMC were incubated in wells of 96-well plates coated with anti-CD3Ab with or without the addition of IL-2, PRL, or the combination of IL-2 and PRL, a significant increase in proliferation (data not shown) as well as increased expression of CD95, CD25 (Figure 6

), and CD71 on the surface of T cells was observed after 72 h regardless of the condition used. When Annexin V binding to CD3^+^ T cells was measured in these cultures, twice as many T cells were apoptotic in cultures containing anti-CD3Ab or anti-CD3Ab+IL-2 compared to T cells cultured in medium clone. In the presence of PRL, apoptosis of T cells activated by anti-CD3Ab or anti-CD3Ab+IL-2 was reduced by approximately 40% (Figure 6). These *ex vivo* results suggested that PRL could protect T cells from activation-induced cell death.

## DISCUSSION

Spontaneous apoptosis of T lymphocytes or T-cell subsets in the circulation of patients with solid tumours is a consistently observed phenomenon (Saito *et al*, 2000; Dworacki *et al*, 2001; Hoffmann *et al*, 2002). Circulating CD8^+^ T lymphocytes appear to be preferentially targeted for apoptosis (Hoffmann *et al*, 2002). Here, we have demonstrated that in postoperative breast cancer patients, a significantly higher proportion of CD3^+^ T cells bind Annexin V and express Fas than that in normal age- and sex-matched controls. The comparison of cord blood samples with normal controls and breast cancer patients showed clearly that without Fas expression on T cells, there was essentially no apoptosis in CD3^+^ lymphocytes. As PBMC of both normal donors and patients with cancer contain antigen-primed T cells, it is not surprising that expression of Fas is detectable on a substantial proportion of these T cells.

Clearly, Fas expression is increased in CD3^+^ cells experiencing chronic antigenic stimulation, as is the case in cancer patients, where Fas expression may be found on virtually all T lymphocytes (Dworacki *et al*, 2001; Hoffmann *et al*, 2002). Nevertheless, Fas expression alone does not necessarily explain higher levels of T-cell apoptosis. The sensitivity of these cells to apoptosis may be influenced by many factors, including the functional state of CD95 (Peter *et al*, 1997) as well as the presence of FasL necessary for crosslinking of the receptor (Ju *et al*, 1995). While the Fas/FasL pathway appears to participate in inducing apoptosis of CD4+ T cells in HIV (Badley *et al*, 1998) and of CD8+ T cells in cancer (Saito *et al*, 2000; Dworacki *et al*, 2001; Hoffmann *et al*, 2002), it is clearly not the only mechanism responsible for elimination of activated T cells. Among other factors that have been suggested are TNF/TNF-R, TRAIL/TRAIL-R (Srivastava, 2001), tumour-derived soluble factors (Taylor *et al*, 2001), and reactive oxygen metabolites (Hansson *et al*, 1999; Schmielau and Finn, 2001). In the case of patients with breast cancer we evaluated, utilisation of sFasL present in the plasma suggests its engagement in Fas-mediated apoptosis. The source of sFasL might be the primary or metastatic breast carcinoma itself. There is evidence in the literature that breast tumours overexpress FasL on the cell surface and that this overexpressed FasL is responsible for T-cell apoptosis (Muschen *et al*, 2000). Of particular interest was the observation that even in patients with stage I disease at diagnosis and no evidence of disease at the time of blood draw, there was a high proportion of T cells undergoing apoptosis. Similar observations are emerging in our studies of patients with other solid tumours (Hoffmann *et al*, 2002). This suggests that tumor-induced or tumour-related effects on the haematopoietic system are long lived. As a result of the small number of patients with advanced disease and no preoperative patients included in this study, we could not evaluate a relation between the stage of disease and magnitude of apoptosis of T lymphocytes. However, the highest proportion of Annexin V^+^Fas^+^ T lymphocytes was observed in the two patients who had metastatic disease at the time of the blood draw. In aggregate, these observations suggest that activated T cells are rapidly turning over in patients with breast cancer and that Fas/FasL interactions may be, in part, responsible for apoptosis of T cells, leading to their extensive turnover. As such rapid turnover is likely to result in the paucity of antitumour effector cells, finding the means of protection of T cells from apoptosis seen in cancer patients is of great interest. Hence, protective effects of PRL on T-cell apoptosis were examined.

Prolactin receptor was found to be expressed on all circulating T cells, and its ligand, PRL, is known to stimulate lymphocyte proliferation (Montgomery *et al*, 1987; Clevenger *et al*, 1990; Yu Lee, 1990; Matera *et al*, 1992) and to protect some lymphoid cells from apoptosis (LaVoie and Witorsch, 1995; Weimann *et al*, 1999). In our hands, exogenous PRL was found to offer protection to T cells from starvation-induced and anti-CD3Ab/IL-2-induced apoptosis but not from death induced by the Fas crosslinking antibody, CH-11. This selective protection of T cells from death by the ubiquitous hormone, PRL, could be envisioned as an attempt to balance haematologic homeostasis, and we expected to find higher plasma PRL levels in patients than controls. However, levels of PRL in plasma were significantly lower in our treated patients with primary operable breast cancer relative to normal controls. This counterintuitive finding was further investigated by excluding premenopausal women from the normal control group for data analysis. Although this manoeuvre eliminated significance of the difference in PRL levels between the cohorts, the patients were still lower than controls. It is possible to speculate that patients have higher serum levels of PRL-binding protein than controls and that lower PRL is related to the apparent lack of protection of T cells from apoptosis in these patients. The observed decreased PRL serum levels in our cohort of patients could also be a result of therapy. However, 11 out of 25 patients were treated with tamoxifen, and it has been shown recently that chronic tamoxifen treatment does not alter PRL plasma levels in postmenopausal women with breast cancer (DeMarinis *et al*, 2000). Furthermore, no difference in Annexin V binding to T cells binding was observed between patients who were treated or not treated with tamoxifen.

Expression of CD25 on CD3^+^ T cells appeared to be related to sensitivity or resistance of the patients' T cells to apoptosis. A much higher percentage of patients' T cells expressed CD25 than that in normal donors (Figure 6) and the level of CD25 expression/cell (MFI) was also higher in patients than controls (Table 4). Surprisingly, our data indicated that expression of CD25 on T cells in patients with breast cancer tended to protect these cells from apoptosis and that CD3^+^CD25^−^ cells preferentially bound Annexin V. Thus, not all circulating T cells were equally sensitive to spontaneous apoptosis in these patients. It has been suggested that IL-2R*α* expression on a subset of T cells (CD4^+^) identifies ‘regulatory’ T lymphocytes (Woo *et al*, 2001). In patients with cancer, the numbers of regulatory cells are increased (Woo *et al*, 2001), and it is possible that they are protected from apoptosis. On the other hand, the IL-2R/IL-2 complex is known to play a crucial role in survival and death of T cells (Van Parijs *et al*, 1997). The presence or absence of IL-2R*α* on the T cell is crucial for its response to IL-2, which may act as a growth factor or a death cytokine depending on the cellular microenvironment (Van Parijs *et al*, 1997). Prolactin, which is known to be able to modulate functions of T lymphocytes and act in concert with IL-2 (Matera *et al*, 1992) is likely to also influence their survival. It is not certain that PRL contributes to protection of CD3^+^CD25^+^ lymphocytes *in vivo*; however, our *ex vivo* experiments suggest that anti-CD3Ab-activated CD25^+^ T cells, which can bind IL-2, are in part protected from AICD by exogenous PRL. The possible role of PRL in protection of activated T cells from apoptosis deserves further studies.
